# A genetic algorithm-based weighted ensemble method for predicting transposon-derived piRNAs

**DOI:** 10.1186/s12859-016-1206-3

**Published:** 2016-08-31

**Authors:** Dingfang Li, Longqiang Luo, Wen Zhang, Feng Liu, Fei Luo

**Affiliations:** 1 School of Mathematics and Statistics, Wuhan University, Wuhan, 430072 China; 2 State Key Lab of Software Engineering, Wuhan University, Wuhan, 430072 China; 3 School of Computer, Wuhan University, Wuhan, 430072 China; 4 International School of Software, Wuhan University, Wuhan, 430072 China

**Keywords:** piRNA, Feature, Genetic algorithm, Ensemble learning

## Abstract

**Background:**

Predicting piwi-interacting RNA (piRNA) is an important topic in the small non-coding RNAs, which provides clues for understanding the generation mechanism of gamete. To the best of our knowledge, several machine learning approaches have been proposed for the piRNA prediction, but there is still room for improvements.

**Results:**

In this paper, we develop a genetic algorithm-based weighted ensemble method for predicting transposon-derived piRNAs. We construct datasets for three species: *Human*, *Mouse* and *Drosophila*. For each species, we compile the balanced dataset and imbalanced dataset, and thus obtain six datasets to build and evaluate prediction models. In the computational experiments, the genetic algorithm-based weighted ensemble method achieves 10-fold cross validation AUC of 0.932, 0.937 and 0.995 on the balanced *Human* dataset, *Mouse* dataset and *Drosophila* dataset, respectively, and achieves AUC of 0.935, 0.939 and 0.996 on the imbalanced datasets of three species. Further, we use the prediction models trained on the *Mouse* dataset to identify piRNAs of other species, and the models demonstrate the good performances in the cross-species prediction.

**Conclusions:**

Compared with other state-of-the-art methods, our method can lead to better performances. In conclusion, the proposed method is promising for the transposon-derived piRNA prediction. The source codes and datasets are available in https://github.com/zw9977129/piRNAPredictor.

**Electronic supplementary material:**

The online version of this article (doi:10.1186/s12859-016-1206-3) contains supplementary material, which is available to authorized users.

## Background

Non-coding RNAs (ncRNAs) are defined as the important functional RNA molecules which are not translated into proteins [1, 2]. According to lengths, ncRNAs are classified into two types: long ncRNAs and short ncRNAs. Usually, long ncRNAs consists of more than 200 nucleotides [3, 4]. Short ncRNAs having 20 ~ 32 nt are defined as small ncRNAs, such as small interfering RNA (siRNA), microRNA (miRNA) and piwi-interacting RNA (piRNA) [5]. piRNA is a distinct class of small ncRNAs expressed in animal cells, especially in germline cells, and the length of piRNA sequences ranges from 26 to 32 in general [6–8]. Compared with miRNA, piRNA lacks conserved secondary structure motifs, and the presence of a 5’ uridine is usually observed in both vertebrates and invertebrates [5, 9, 10].

piRNAs play an important role in the transposon silencing [11–15]. About nearly one-third of the fruit fly and one-half of human genomes are transposon elements. These transposons move within the genome and induce insertions, deletions, and mutations, which may cause the genome instability. piRNA pathway is an important genome defense mechanism to maintain genome integrity. Loaded into PIWI proteins, piRNAs serve as a guide to target the transposon transcripts by sequence complementarity with mismatches, and then the transposon transcripts will be cleaved and degraded, producing secondary piRNAs, which is called ping-pong cycle in fruit fly [13–17]. Therefore, predicting transposon-derived piRNAs provides biological significance and insights into the piRNA pathway.

The wet method utilizes immunoprecipitation and deep sequencing to identify piRNAs [18]. Since piRNAs are diverse and non-conserved, wet methods are time-consuming and costly [5, 9, 10]. Since the development of information science, the piRNA prediction based on the known data becomes an alternative. As far as we know, several computational methods have been proposed for piRNA prediction. Betel et al. developed the position-specific usage method to recognize piRNAs [19]. Zhang et al. utilized a *k*-mer feature, and adopted support vector machine (SVM) to build the classifier (named piRNApredictor) for piRNA prediction [20]. Wang et al. proposed a method named Piano to predict piRNAs, by using piRNA-transposon interaction information and SVM [21]. These methods exploited different features of piRNAs, and build the prediction models by using machine learning methods.

Motivated by previous works, we attempt to differentiate transposon-derived piRNAs from non-piRNAs based on the sequential and physicochemical features. As far as we know, there are several critical issues for developing high-accuracy models. Firstly, the accuracy of models is highly dependent on the diversity of features. In order to achieve high-accuracy models, we should consider as many sequence-derived features as possible. Secondly, how to effectively combine various features for high-accuracy models is very challenging. In the previous work [22], we adopted the exhaustive search strategy to combine five sequence-derived features to predict piRNAs, and used the AUC scores of individual feature-based models as weights in the ensemble learning. However, the method can’t effectively integrate a great amount of features (NP-hard complexity: 2^*N*^-1 combinations of features, *N* is the number of features), and the determination of weights is arbitrary.

In this paper, we develop a genetic algorithm-based weighted ensemble method (GA-WE) to effectively integrate twenty-three discriminative features for the piRNA prediction. Specifically, individual features-based models are constructed as base learners, and the weighted average of their outputs is adopted as the final scores in the stage of prediction. Genetic algorithm (GA) is to search for the optimal weights for the base learners. Moreover, the proposed method can determine the weights for each base learner in a self-tune manner.

We construct datasets for three species: *Human*, *Mouse* and *Drosophila*. For each species, we compile the balanced dataset and imbalanced dataset, and thus obtain six datasets to build and evaluate prediction models. In the 10-fold cross validation experiments, the GA-WE method achieves AUC of 0.932, 0.937 and 0.995 on the balanced *Human* dataset, *Mouse* dataset and *Drosophila* dataset, respectively, and achieves AUC of 0.935, 0.939 and 0.996 on the imbalanced datasets of three species. Further, we use the prediction models trained on the *Mouse* dataset to identify piRNAs of other species. The results demonstrate that the models can produce good performances in the cross-species prediction. Compared with other state-of-the-art methods, our method produces better performances as well as good robustness. Therefore, the proposed method is promising for the transposon-derived piRNA prediction.

## Methods

### Datasets

In this paper, we construct datasets for three species: *Human*, *Mouse* and *Drosophila*, and use them to build prediction models and make evaluations.

As shown in Table 1, raw real piRNAs, raw non-piRNA ncRNAs and transposons are downloaded from NONCODE version 3.0 [23], UCSC Genome Browser [24] and NCBI Gene Expression Omnibus [18, 25]. NONCODE is an integrated knowledge database about non-coding RNAs [23]. The UCSC Genome Browser is an interactive website offering access to genome sequence data from a variety of vertebrate and invertebrate species, integrated with a large collection of aligned annotations [24]. The NCBI Gene Expression Omnibus is the largest fully public repository for high-throughput molecular abundance data, primarily gene expression data [18, 25].

**Table 1:** Raw data about three species

Species	Raw real piRNAs	Raw non-piRNA ncRNAs	Transposons
*Human*	32,152 (NONCODE v3.0)	59,003 (NONCODE v3.0)	4,679,772 (UCSC, hg38)
*Mouse*	75,814 (NONCODE v3.0)	43,855 (NONCODE v3.0)	3,660,356 (UCSC, mm10)
*Drosophila*	12,903 (NCBI, GSE9138)	102,655 (NONCODE v3.0)	37,326 (UCSC, dm6)

The datasets are compiled from the raw data (Table 1). By aligning raw real piRNAs to transposons with SeqMap (three mismatches at most) [26], the aligned real piRNAs are transposon-matched piRNAs, and they are adopted as the set of real piRNAs. The length of real piRNAs ranges from 16 to 35. To meet the length range of real piRNAs, we remove non-piRNA ncRNAs shorter than 16, and cut non-piRNA ncRNAs longer than 35 by simulating length distribution of real piRNAs. The cut sequences are then aligned to transposons, and the aligned ones are used as the set of pseudo piRNAs. The real piRNAs and the pseudo piRNAs for three species are shown in Table 2. In order to the build prediction models, we build the datasets based on real piRNAs and pseudo piRNAs. To avoid the data bias caused by different size of positive instances and negative instances, we construct both balanced datasets and imbalanced datasets for three species. For balanced datasets, all real piRNAs are adopted as positive instances, and we sample the same number of pseudo piRNAs as negative instances. For imbalanced datasets, we use all real piRNAs and pseudo piRNAs as positive instances and negative instances.

**Table 2:** Number of real piRNAs and pseudo piRNA

Species	Real piRNAs	Pseudo piRNA
*Human*	7,405	21,846
*Mouse*	13,998	40,712
*Drosophila*	9,214	22,855

### Features of piRNAs

For prediction, we should explore informative features that can characterize piRNAs and convert variable-length piRNA sequences into fixed-length feature vectors. Here, we consider various potential features that are widely used in biological sequence prediction. Among these features, six features have been utilized for the piRNA prediction, while the rest are taken into account for the first time. These sequence-derived features are briefly introduced as follows.

Spectrum profile: *k*-spectrum profile, also named *k*-mer feature, is to count the occurrences of *k*-mers (*k*-length contiguous strings) in sequences (*k* ≥ 1), and its success has been proved by numerous bioinformatics applications [27–30].

Mismatch profile: (*k*, *m*)-mismatch profile also counts the occurrences of *k*-mers, but allows max *m* (*m* < *k*) inexact matching, which is the penalization of spectrum profile [30, 31].

Subsequence profile: (*k*, *w*)-subsequence profile considers not only the contiguous *k*-mers but also the non- contiguous *k*-mers, and the penalty factor *w* (0 ≤ *w* ≤ 1) is used to penalize the gap of non-contiguous *k*-mers [30, 32].

Reverse compliment *k*-mer (*k*-RevcKmer): *k*-RevcKmer is a variant of the basic *k-*mer, in which the *k*-mers are not expected to be strand-specific [29, 33, 34].

Parallel correlation pseudo dinucleotide composition (PCPseDNC): PCPseDNC is proposed to avoid losing the physicochemical properties of dinucleotides. PCPseDNC of a sequence consists of two components, the first component represents the occurrences of different dinucleotides, while the other component reflects the physicochemical properties of dinucleotides [28, 29, 35].

Three features: parallel correlation pseudo trinucleotide composition (PCPseTNC), series correlation pseudo dinucleotide composition (SCPseDNC) and series correlation pseudo trinucleotide composition (SCPseTNC) are similar to the PCPseDNC. PCPseTNC considers the occurrences of trinucleotides and their physicochemical properties, and SCPseDNC and SCPseTNC consider series correlations of physicochemical properties of dinucleotides or trinucleotides [28, 29, 35, 36].

Sparse profile [37] and position-specific scoring matrix (PSSM) [38–40] are usually generated from the fixed-length sequences. Here, we use a simple strategy to process the variable-length sequences, and obtain the features. We truncate the first *d* nucleotides of long sequences which lengths are more than *d*, and extend short sequences which lengths are less than *d* by adding the null character. Here, ‘*E*’ represent the null character, which are added to the short sequences to meet the length *d.* In this way, all variable-length sequences are converted into fixed-length sequences, and the fixed-length sequences consist of five letters {*A*, *C*, *G*, *T*, *E*}. For the sparse profile, by encoding each letter of sequence as a 5*-*bit vector with 4 bits set to zero and 1 bit set to one, the sparse profile of a sequence is obtained by merging the bit vector for its letters. For the PSSM feature, PSSM can be calculated on the fixed-length sequences consisted of five letters {*A*, *C*, *G*, *T*, *E*} [38–40]. Given a new sequence, it is truncated or extended, and then is encoded by PSSM as the feature vector. The PSSM representation of sequence *x* = *R*_1_*R*_2_ … *R*_*d*_ is defined as:

$$ {f_d}^{PSSM}(x)=\left( score\;\left({R}_1\right),\; score\left({R}_2\right),\dots,\;score\left({R}_d\right)\right) $$

where

$$ score\left({R}_k\right)=\left\{\begin{array}{l}m\left({R}_k\right),\kern0.75em {R}_k\in \left\{A,C,G,T\right\}\\ {}0,\kern2.75em {R}_k=E\end{array}\right.,k=1,2,\dots, d $$

and *m*(*R*_*k*_) represents the score of *R*_*k*_ in the *k*-th column of PSSM, if *R*_*k*_ ∈ {*A*, *C*, *G*, *T*}, *k* = 1, 2, …, *d*.

Local structure-sequence triplet elements (LSSTE): LSSTE adopts the piRNA-transposon interaction information to extract 32 different triplet elements, which contain the structural information of transposon-piRNA alignment as well as the piRNA sequence information [21, 41, 42].

A total of twenty-three feature vectors are finally obtained, and they are summarized in Table 3.

**Table 3:** Twenty-three sequence-derived features

Index	Feature	Dimension	Parameter	Annotation
F1	1-Spectrum Profile	4	No Parameters	Used in [20]
F2	2-Spectrum Profile	16	No Parameters	Used in [20]
F3	3-Spectrum Profile	64	No Parameters	Used in [20]
F4	4-Spectrum Profile	256	No Parameters	Used in [20]
F5	5-Spectrum Profile	1024	No Parameters	Used in [20]
F6	(3, *m*)-mismatch profile	64	*m*: the max mismatches	New features
F7	(4, *m*)-mismatch profile	256	*m*: the max mismatches	New features
F8	(5, *m*)-mismatch profile	1024	*m*: the max mismatches	New features
F9	(3, *w*)-subsequence profile	64	*w*: penalty for the non-contiguous matching	New features
F10	(4, *w*)-subsequence profile	256	*w*: penalty for the non-contiguous matching	New features
F11	(5, *w*)-subsequence profile	1024	*w*: penalty for the non-contiguous matching	New features
F12	1-RevcKmer	2	No Parameters	New features
F13	2-RevcKmer	10	No Parameters	New features
F14	3-RevcKmer	32	No Parameters	New features
F15	4-RevcKmer	136	No Parameters	New features
F16	5-RevcKmer	528	No Parameters	New features
F17	PCPseDNC	16 + *λ*	*λ*: the highest counted rank of the correlation	New features
F18	PCPseTNC	64 + *λ*	*λ*: the highest counted rank of the correlation	New features
F19	SCPseDNC	16 + 6 × *λ*	*λ*: the highest counted rank of the correlation	New features
F20	SCPseTNC	64 + 12 × *λ*	*λ*: the highest counted rank of the correlation	New features
F21	Sparse Profile	5 × *d*	*d*: the fixed length of sequences	New features
F22	PSSM	*d*	*d*: the fixed length of sequences	New features
F23	LSSTE	32	No parameters	Used in [21]

### The GA-based weighted ensemble method

In the view of information science, a variety of features can bring diverse information, and the combination of various features can lead to better performance than individual features [22, 43–46]. Ensemble learning is a sophisticated feature combination technique widely used in bioinformatics. Its success has been proved by numerous bioinformatics applications, such as the prediction of B-cell epitopes [44] and the prediction of immunogenic T-cell epitopes [45].

To the best of our knowledge, there are two crucial issues for designing good ensemble systems, i.e. base learners and combination rules. First, the training sequences are encoded into different feature vectors, respectively, and multiple base learners are constructed on these feature vectors by using classification engines. We compare two most popular classification methods, random forest (RF) [47] and support vector machine (SVM) [48] (results are given in the section ‘Results and Discussion’), and finally adopt RF as the basic classification engine because of its high efficiency and high accuracy. Then, how to combine the outputs of base learners is crucial for the success of our ensemble system. Our ensemble learning adopts the weighted average of outputs from base learners as the final score. However, the determination of weights is difficult. In this paper, we develop a genetic algorithm (GA)-based weighted ensemble method, which can automatically determine the optimal weights and construct high-accuracy prediction models.

Given *N* features, we can construct *N* base learners: *f*_1_, *f*_2_, …, *f*_*N*_ on training set. *w*_1_, *w*_2_, …, *w*_*N*_ (∑_*i* = 1_^*N*^*w*_*i*_, 0 ≤ *w*_*i*_ ≤ 1, *i* = 1, 2, …, *N*) represent the corresponding weights. For a testing sequence *x*, *f*_*i*_(*x*) ∈ [0, 1] represents the probability of predicting *x* as real piRNA, *i* = 1, 2, …, *N*, and the final predicted results of the weighted ensemble model is given as:

$$ F(x)={\displaystyle {\sum}_{i=1}^N{w}_i{f}_i(x)} $$

As discussed above, the optimal weights are very important for the weighted ensemble model. We consider the determination of weights as an optimization problem and adopt the genetic algorithm (GA) to search the optimal weights. GA is a search approach that simulates the process of natural selection. It can effectively search the interesting space and easily solve complex problems without requiring the prior knowledge about the space. Here, we use the adaptive genetic algorithm [49]. In the adaptive genetic algorithm, crossover probability and mutation probability are dynamically adjusted according to the fitness scores of chromosomes. The size of an initial population is 1000 chromosomes, and the iteration number is 500.

**Fig. 1:**
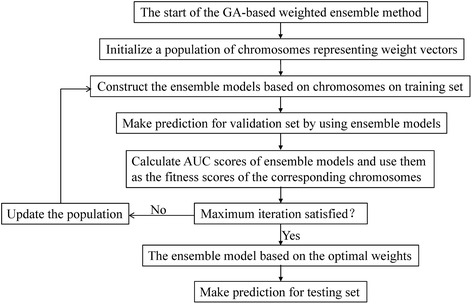
Flowchart of the GA-based weighted ensemble method

The flowchart of the GA-WE method is shown in Fig. 1. In each training-testing process, the dataset is split into the training set, the validation set and the testing set. In the GA optimization, a chromosome represents weights. For each chromosome (weights), the weighted ensemble model is constructed on the training set, and makes predictions for the validation set. The AUC score of the weighted ensemble model on the validation set is taken as the fitness of the chromosome. After randomly generating an initial population, the population is updated by three operators: selection, crossover and mutation, and the best individual with a chromosome will be obtained. Finally, the weighted ensemble model with the optimal weights is used to make predictions for the testing set.

## Results and discussion

### Performance evaluation metrics

The proposed methods are evaluated by the 10-fold cross validation (10-CV). In the 10-CV, a dataset is randomly split into 10 subsets with equal size. For each round of 10-CV, 8 subsets are used as the training set, 1 subset is used as the validation set and the rest one is considered as the testing set. Prediction models are constructed on the training set, and the parameters or optimal weights of models are determined on the validation set. Finally, optimized prediction models are adopted to predict the testing set. This processing is repeated until all subsets are ever used for testing.

Here, we adopt several metrics to assess the performances of prediction models, including the accuracy (ACC), sensitivity (SN), specificity (SP) and the AUC score (the area under the ROC curve). These metrics are defined as:

$$ SN=\frac{TP}{TP+FN} $$

$$ SP=\frac{TN}{TN+FP} $$

$$ ACC=\frac{TP+TN}{TP+TN+FP+FN} $$

Where TP, FP, TN and FN are the numbers of true positives, false positives, true negatives and false negatives, respectively. The ROC curve is plotted by using the false positive rate (1-specificity) against the true positive rate (sensitivity) for different cutoff thresholds. Here, we consider the AUC as the primary metric, for it assesses the performance regardless of any threshold.

### Parameters of various features

**Fig. 2:**
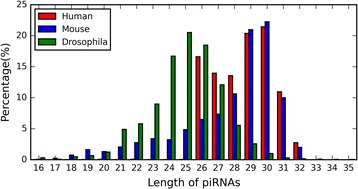
The length distribution of piRNAs in three species (*Human*, *Mouse* and *Drosophila*)

As shown in Table 3, we consider twenty-three sequence-derived features to develop prediction models. Since subsequence profile, PCPseDNC, PCPseTNC, SCPseDNC, SCPseTNC, sparse profile and PSSM have parameters, we discuss how to determine the parameters based on the balanced *Human* dataset, and use them in the following studies. Considering the parameter *λ* and *d* refer to the length of piRNAs, we analyze the length distribution of piRNAs in three species (*Human*, *Mouse* and *Drosophila*). As shown in Fig. 2, the length of piRNAs ranges from 16 to 35, and reaches the peak at 30 for *Human* and *Mouse*, and 25 for *Drosophila*. Here, the impacts of parameters are evaluated according to the 10-CV performances of corresponding models.

In the mismatch profile, the parameter *m* represents the max mismatches. Here, we assume that *m* does not exceed one third of length of *k*-mers. Therefore, (3, 1)-mismatch profile, (4, 1)-mismatch profile and (5, 1)-mismatch profile are used.

**Fig. 3:**
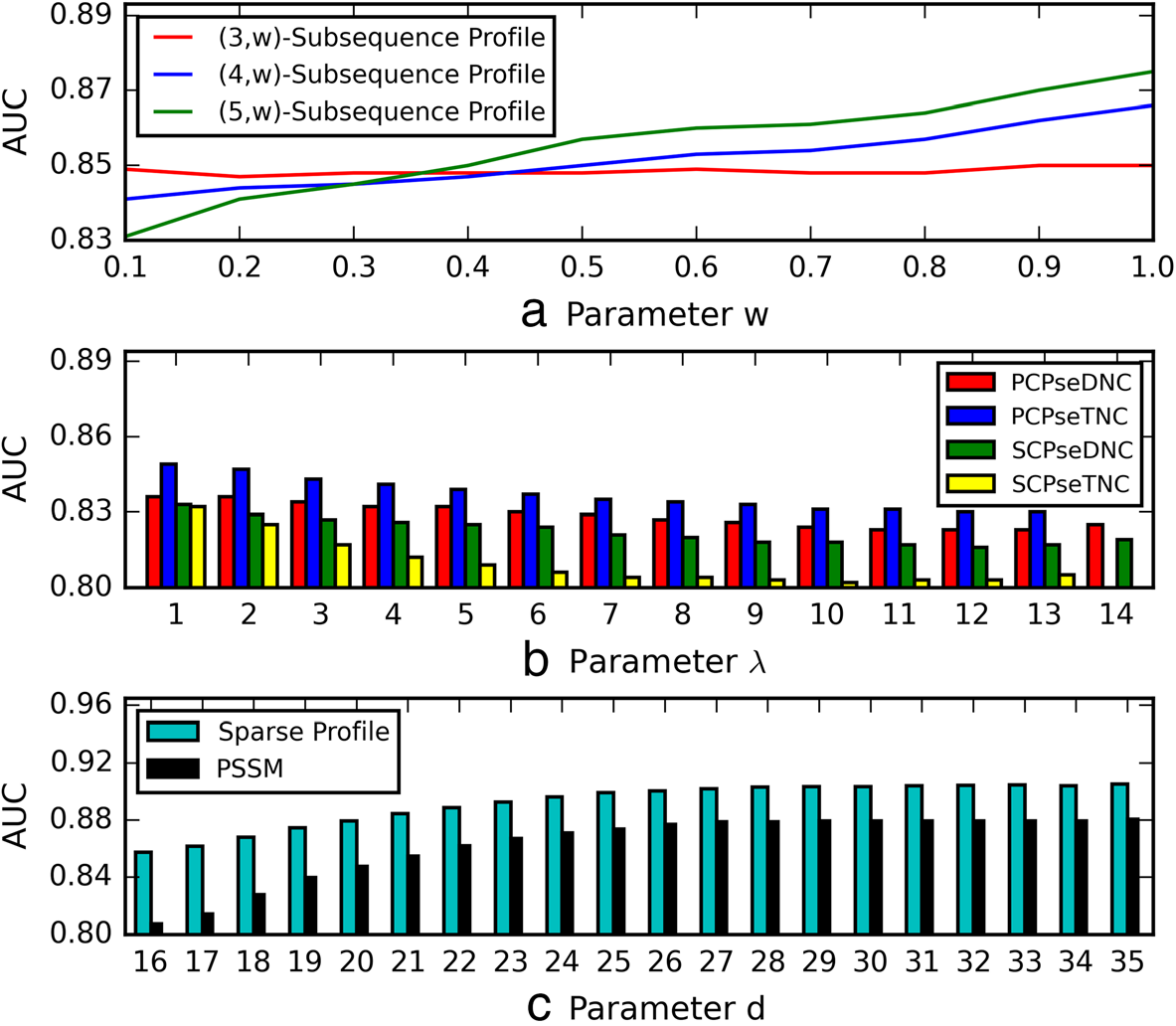
**a** AUC scores of the (*k*, *w*)-subsequence profile-based models with the variation of parameter *w* on balanced *Human* dataset; **b** AUC scores of the PCPseDNC, PCPseTNC, SCPseDNC and SCPseTNC-based models with the variation of the parameter *λ* on balanced *Human* dataset; **c** AUC scores of the sparse profile and PSSM-based models with the variation of the parameter *d* on balanced *Human* dataset

In the subsequence profile, the parameter *w* represents the gap penalty of non-contiguous *k*-mers. As shown in Fig. 3 (a), *w* = 1 produces the best AUC scores for (3, *w*)-subsequence profile, (4, *w*)- subsequence profile and (5, *w*)-subsequence profile. Therefore, (3, 1)-subsequence profile, (4, 1)-subsequence profile and (5, 1)-subsequence profile are finally adopted in the following study.

In the PCPseDNC, PCPseTNC, SCPseDNC and SCPseTNC, the parameter *λ* represents the highest counted rank of the correlation, 1 ≤ *λ* ≤ *L* − 2 (for the PCPseDNC and SCPseDNC); 1 ≤ *λ* ≤ *L* − 3 (for the PCPseTNC and SCPseTNC) [28, 29, 35, 36]. *L* is the length of shortest piRNA sequences, and is 16 according to Fig. 2. To test the impact of the parameter *λ* on the four features, we construct the prediction models by using different values. As shown in Fig. 3 (b). *λ* = 1 leads to the best AUC scores for PCPseDNC, PCPseTNC, SCPseDNC and SCPseTNC. Therefore, the best parameters are adopted for the final prediction models.

In the sparse profile and PSSM, the parameter *d* represents the fixed length of sequences. As show in Fig. 2, the lengths of piRNAs range from 16 to 35. Therefore, the prediction models are constructed based on different length*s*. As shown in Fig. 3 (c), *d* = 35 produces the best AUC scores for the sparse profile and PSSM feature. Therefore, we set the parameter *d* as 35 for the sparse profile feature and the PSSM feature.

### Evaluation of various features

After discussing feature parameters, we compare the capabilities of various features for the piRNA prediction. Here, individual feature-based models are constructed on balanced *Human* dataset and imbalanced *Human* dataset by using classification engines, and the performances of these models are evaluated by 10-CV.

To test different classifiers, we respectively adopt the random forest (RF) and support vector machine (SVM) to build the individual feature-based prediction models. Here, we use the python package “scikit-learn” to implement RF and SVM, and default values are adopted for parameters. The results demonstrate that RF can produce better performances in most cases (13 out of the 23 individual feature-based models). Moreover, RF runs much faster than SVM, and it is very important for implementing the following experiments. Results of RF models and SVM models are provided in the Additional files 1 and 2. For these reasons, RF is adopted in the following study.

To test the impacts of the ratio of positive instances versus negative instances, we build the individual feature-based prediction models based on the balanced human datasets and the imbalanced human dataset. As shown in Table 4 and Table 5, the prediction models produce similar results on the balanced dataset and imbalanced dataset, indicating that they are robust to the different datasets. The performances of individual feature-based models help to rank the importance of features. According to Table 4 and Table 5, the sparse profile yields the best results among these features, and the performance of LSSTE is much poorer than that of other features. Therefore, we adopt features indexed from F1 to F22 (“F1 ~ F22”) for the final ensemble models.

**Table 4:** The performances of individual feature-based models on balanced *Human* dataset

Index	Feature	AUC	ACC	SN	SP
F1	1-Spectrum Profile	0.754	0.690	0.731	0.649
F2	2-Spectrum Profile	0.841	0.756	0.780	0.732
F3	3-Spectrum Profile	0.839	0.750	0.747	0.754
F4	4-Spectrum Profile	0.829	0.740	0.732	0.748
F5	5-Spectrum Profile	0.802	0.718	0.681	0.755
F6	(3,1)-Mismatch Profile	0.862	0.772	0.819	0.725
F7	(4,1)-Mismatch Profile	0.854	0.761	0.788	0.734
F8	(5,1)-Mismatch Profile	0.842	0.750	0.754	0.747
F9	(3,1)-Subsequence Profile	0.850	0.767	0.809	0.725
F10	(4,1)-Subsequence Profile	0.866	0.782	0.821	0.743
F11	(5,1)-Subsequence Profile	0.875	0.791	0.829	0.754
F12	1-RevcKmer	0.746	0.699	0.889	0.509
F13	2-RevcKmer	0.803	0.724	0.774	0.673
F14	3-RevcKmer	0.818	0.732	0.765	0.698
F15	4-RevcKmer	0.808	0.718	0.717	0.718
F16	5-RevcKmer	0.791	0.702	0.658	0.746
F17	PCPseDNC	0.836	0.757	0.776	0.738
F18	PCPseTNC	0.849	0.765	0.787	0.742
F19	SCPseDNC	0.833	0.754	0.770	0.739
F20	SCPseTNC	0.832	0.751	0.777	0.725
F21	Sparse Profile	0.904	0.819	0.815	0.824
F22	PSSM	0.880	0.807	0.815	0.799
F23	LSSTE	0.688	0.631	0.664	0.598

**Table 5:** The performances of individual feature-based models on imbalanced *Human* dataset

Index	Feature	AUC	ACC	SN	SP
F1	1-Spectrum Profile	0.748	0.739	0.398	0.854
F2	2-Spectrum Profile	0.841	0.808	0.416	0.940
F3	3-Spectrum Profile	0.850	0.814	0.321	0.982
F4	4-Spectrum Profile	0.844	0.811	0.284	0.989
F5	5-Spectrum Profile	0.836	0.813	0.305	0.986
F6	(3,1)-Mismatch Profile	0.867	0.824	0.427	0.959
F7	(4,1)-Mismatch Profile	0.856	0.814	0.328	0.979
F8	(5,1)-Mismatch Profile	0.851	0.810	0.277	0.991
F9	(3,1)-Subsequence Profile	0.850	0.808	0.443	0.932
F10	(4,1)-Subsequence Profile	0.864	0.822	0.473	0.940
F11	(5,1)-Subsequence Profile	0.871	0.829	0.492	0.944
F12	1-RevcKmer	0.745	0.746	0.005	0.997
F13	2-RevcKmer	0.803	0.778	0.411	0.902
F14	3-RevcKmer	0.823	0.800	0.265	0.981
F15	4-RevcKmer	0.823	0.803	0.241	0.993
F16	5-RevcKmer	0.818	0.806	0.255	0.992
F17	PCPseDNC	0.841	0.806	0.374	0.952
F18	PCPseTNC	0.857	0.813	0.337	0.975
F19	SCPseDNC	0.836	0.803	0.346	0.958
F20	SCPseTNC	0.842	0.808	0.312	0.977
F21	Sparse Profile	0.905	0.856	0.634	0.932
F22	PSSM	0.882	0.832	0.584	0.916
F23	LSSTE	0.688	0.766	0.175	0.966

### Performances of GA-based weighted ensemble method

The GA-based weighted ensemble (GA-WE) method integrates sequence-derived features and constructs high-accuracy prediction models. We evaluate the performances of the GA-WE model on the datasets of three species. Moreover, we carry out the cross-species prediction, in which we build prediction models on *Mouse* species, and make prediction for other species.

#### Results of GA-WE models on three species

As show in Table 6, the GA-WE models achieve AUC of 0.932, accuracy of 0.839, sensitivity of 0.858 and specificity of 0.820 on the balanced *Human* dataset. Compared with the best individual features-based model (the sparse profile-based model), the GA-WE model improves AUC of >3%, indicating the GA-WE model can effectively combine various features to enhance performances. The proposed method also performs accurate prediction on balanced *Mouse* dataset, achieving AUC of 0.937. Compared with the piRNA prediction on mammalian: *Human* and *Mouse*, the prediction on *Drosophila* is much better, achieving AUC of 0.995. Similarly, the GA-WE model performs high-accuracy prediction on the imbalanced datasets of the three species, achieves AUC of 0.935, 0.939 and 0.996, respectively, which demonstrates that the GA-WE model has not only high accuracy but also good robustness.

**Table 6:** The performances of the GA-WE model on three species (*Human*, *Mouse* and *Drosophila*)

Dataset	Species	AUC	ACC	SN	SP
Balanced	*Human*	0.932	0.839	0.858	0.820
	*Mouse*	0.937	0.838	0.824	0.852
	*Drosophila*	0.995	0.959	0.951	0.966
Imbalanced	*Human*	0.935	0.869	0.687	0.931
	*Mouse*	0.939	0.889	0.745	0.939
	*Drosophila*	0.996	0.958	0.897	0.983

**Fig. 4:**
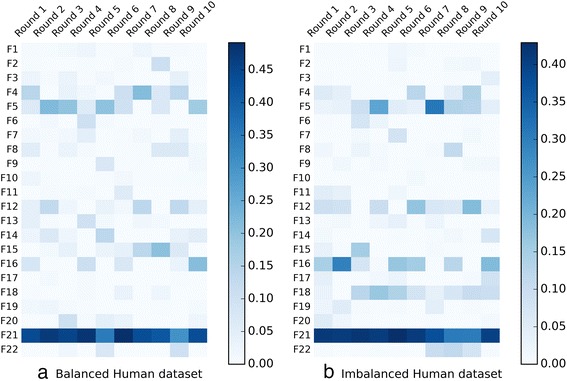
Optimal weights for the GA-WE model in each fold of 10-CV

Further, we investigate the optimal weights for the GA-WE model in each fold of 10-CV. Taking *Human* dataset as an example, the optimal weights of “F1 ~ F22” for the GA-WE model are visualized by the heat map (Fig. 4). We can draw several conclusions from the results. Firstly, different features have different weights in each fold of 10-CV, and the optimal weights can lead to the best ensemble model. Secondly, optimal weights reflect the contributions of the corresponding features for the ensemble model, and the feature having the best performances for piRNA prediction always makes the greatest contribution to the ensemble model. For example, the sparse profile (F21) performs the highest contribution to the ensemble model in each fold of 10-CV, for the sparse profile has the best predictive ability among all features. Thirdly, the optimal weights for the ensemble model depend on the training set, and determining the optimal weights is necessary for building high-accuracy models.

#### Results of cross-species prediction

Considering that *Mouse* instances are more than *Human* instances and *Drosophila* instances, we construct the GA-WE model on *Mouse* dataset, and make predictions for *Human* dataset and *Drosophila* dataset.

As shown in Table 7, the GA-WE model trained with *Mouse* dataset achieves AUC of 0.863 and 0.687 on the balanced *Human* and *Drosophila* datasets, and achieves AUC of 0.868 and 0.746 on the imbalanced datasets of the two species. Compared with the experiments on a same species, the cross-species experiments produce lower scores, indicating that piRNAs derived from different species may have different patterns. Moreover, the results on *Human* dataset are better than the results on *Drosophila* dataset, and the possible reason is that the length distribution of *Mouse* piRNAs is similar to that of *Human* piRNAs, and is different from that of *Drosophila* piRNAs (shown in Fig. 2). Therefore, we’d better train models and make predictions based on a same species.

**Table 7:** The performances of cross-species prediction

Dataset	Species	AUC	ACC	SN	SP
Balanced	*Human*	0.863	0.788	0.796	0.781
	*Drosophila*	0.687	0.668	0.639	0.698
Imbalanced	*Human*	0.868	0.811	0.425	0.942
	*Drosophila*	0.746	0.774	0.370	0.936

### Comparison with other state-of-the-art methods

Here, three latest methods: piRNApredictor [20], Piano [21] and our previous work [22] are adopted as the benchmark methods, for they build prediction models based on machine learning methods. piRNApredictor used *k*-mer feature (i.e, spectrum profile), *k* = 1, 2, 3, 4, 5, and Piano used the LSSTE feature. piRNApredictor and Piano adopted the support vector machine (SVM) to construct prediction models. Our previous work adopted the exhaustive search strategy to combine five sequence-derived features to predict piRNAs. We implement piRNApredictor obtain the results. Since the source codes of Piano are available at http://ento.njau.edu.cn/Piano.html, we can run the program on the benchmark datasets. The proposed methods and three benchmark methods are evaluated on six benchmark datasets by using 10-CV.

As shown in Table 8, our previous work, piRNApredictor and Piano achieve AUC of 0.920, 0.894 and 0.592 on the balanced *Human* dataset, respectively. Our GA-WE model produces AUC of 0.932 on the dataset. The proposed method also yields much better performances than piRNApredictor and Piano on the balanced *Mouse* dataset and balanced *Drosophila* dataset. There are several reasons for the superior performances of our method. Firstly, various useful features can guarantee the diversity for the GA-WE model. Secondly, the GA-WE model automatically determines the optimal weights on validation set.

**Table 8:** Performances of GA-WE and the state-of-the-art methods on three species

Dataset	Species	Method	AUC	ACC	SN	SP
Balanced	*Human*	Piano	0.592	0.560	0.855	0.265
		piRNApredictor	0.894	0.812	0.859	0.764
		Ensemble Learning	0.920	0.807	0.815	0.800
		GA-WE	0.932	0.839	0.858	0.820
	*Mouse*	Piano	0.445	0.5365	0.837	0.236
		piRNApredictor	0.892	0.819	0.862	0.776
		Ensemble Learning	0.924	0.810	0.863	0.756
		GA-WE	0.937	0.838	0.826	0.850
	*Drosophila*	Piano	0.741	0.692	0.836	0.547
		piRNApredictor	0.983	0.952	0.927	0.977
		Ensemble Learning	0.994	0.958	0.952	0.965
		GA-WE	0.995	0.959	0.949	0.966
Imbalanced	*Human*	Piano	0.449	0.747	0.000	1.000
		piRNApredictor	0.905	0.847	0.548	0.949
		Ensemble Learning	0.922	0.836	0.589	0.919
		GA-WE	0.935	0.869	0.687	0.931
	*Mouse*	Piano	0.441	0.744	0.000	1.000
		piRNApredictor	0.892	0.848	0.568	0.944
		Ensemble Learning	0.928	0.849	0.586	0.940
		GA-WE	0.939	0.889	0.745	0.939
	*Drosophila*	Piano	0.804	0.712	0.000	1.000
		piRNApredictor	0.982	0.961	0.902	0.985
		Ensemble Learning	0.995	0.965	0.920	0.984
		GA-WE	0.996	0.964	0.940	0.973

Further, we compare the capabilities of the GA-WE method with the state-of-the-art methods in the cross-species prediction. All models are constructed on *Mouse* dataset, and make predictions for *Human* and *Drosophila* dataset. As shown in Table 9, our GA-WE model trained with *Mouse* dataset performs better than the state-of-the-art methods on the *Human* datasets, but performs worse than piRNApredictor on the *Drosophila* dataset. Moreover, the performances on *Human* dataset are always better than that on *Drosophila* dataset regardless of any method, and the possible reason is that the length distribution of *Mouse* piRNAs is similar to that of *Human* piRNAs, and is different from that of *Drosophila* piRNAs (shown in Fig. 2). In general, our method can produce satisfying results in the cross-species prediction.

**Table 9:** Performances of GA-WE and the state-of-the-art methods in the cross-species prediction

Dataset	Species	Method	AUC	ACC	SN	SP
Balanced	*Human*	Piano	0.431	0.558	0.878	0.238
		piRNApredictor	0.850	0.783	0.781	0.784
		Ensemble Learning	0.845	0.774	0.764	0.784
		GA-WE	0.863	0.788	0.796	0.781
	*Drosophila*	Piano	0.367	0.587	0.905	0.270
		piRNApredictor	0.728	0.650	0.630	0.669
		Ensemble Learning	0.682	0.628	0.512	0.745
		GA-WE	0.687	0.668	0.639	0.698
Imbalanced	*Human*	Piano	0.426	0.747	0.000	1.000
		piRNApredictor	0.856	0.823	0.507	0.931
		Ensemble Learning	0.856	0.783	0.300	0.946
		GA-WE	0.868	0.811	0.425	0.942
	*Drosophila*	Piano	0.369	0.713	0.000	1.000
		piRNApredictor	0.783	0.773	0.422	0.915
		Ensemble Learning	0.750	0.736	0.275	0.921
		GA-WE	0.746	0.774	0.370	0.936

## Conclusions

In this paper, we develop the GA-based weighted ensemble method, which can automatically determine the importance of different information resources and produce high-accuracy performances. We compile the *Human*, *Mouse* and *Drosophila* datasets from NONCODE version 3.0, UCSC Genome Browser and NCBI Gene Expression Omnibus. In the computational experiments, the GA-based weighted ensemble method achieves AUC of >93% by 10-CV. Compared with other state-of-the-art methods, our method produces better performances as well as good robustness. In conclusion, the proposed method is promising for transposon-derived piRNA prediction. The source codes and datasets are available in https://github.com/zw9977129/piRNAPredictor.

## Additional file

Additional file 1: Table S1. The performances of RF models and SVM models on the balanced Human dataset. (XLSX 13 kb) Additional file 2: Table S2. The performances of RF models and SVM models on the imbalanced Human dataset. (XLSX 13 kb)

## Abbreviations

“F1~F22”: The features indexed from F1 to F22
10-CV: 10-fold cross validation
ACC: Accuracy
AUC: Area under ROC curve
GA: Genetic algorithm
GA-WE: Genetic algorithm-based weighted ensemble
LSSTE: Local structure-sequence triplet elements
PCPseDNC: Parallel correlation pseudo dinucleotide composition
PCPseTNC: Parallel correlation pseudo trinucleotide composition
PSSM: Position-specific scoring matrix
RF: Random forest
SCPseDNC: Series correlation pseudo dinucleotide composition
SCPseTNC: Series correlation pseudo trinucleotide composition
SN: Sensitivity
SP: Specificity
SVM: Support vector machine
